# Impact of Neoadjuvant Chemotherapy (NAC) on Biomarker Expression in Breast Cancer

**DOI:** 10.3390/medicina60050737

**Published:** 2024-04-29

**Authors:** Suji Lee, Jee Yeon Kim, So Jeong Lee, Chung Su Hwang, Hyun Jung Lee, Kyung Bin Kim, Jung Hee Lee, Dong Hoon Shin, Kyung Un Choi, Chang Hun Lee, Gi Yeong Huh, Ahrong Kim

**Affiliations:** 1 Department of Pathology, Pusan National University Hospital, Biomedical Research Institution, 179 Gudeok-ro, Seo-gu, Busan 49241, Republic of Korea; 2 Department of Pathology, School of Medicine, Pusan National University, Beomeori, Mulgeum-eop, Yangsan 50612, Republic of Korea; 3 Department of Pathology, Yangsan Pusan National University Hospital, Medical Research Institute, Beomeori, Mulgeum-eop, Yangsan 50612, Republic of Korea; 4 Department of Pathology, Seegene Medial Foundation Busan, Joongangdaero 297, Busan 48792, Republic of Korea

**Keywords:** breast cancers, neoadjuvant chemotherapy, breast cancer subtype, biomarker, p53, prognostic factor

## Abstract

*Background and Objectives*: This study aimed to explore biomarker change after NAC (neoadjuvant chemotherapy) and to investigate biomarker expression as a prognostic factor in patients with residual disease (RD) after NAC. *Materials and Methods*: We retrospectively evaluated 104 patients with invasive breast cancer, who underwent NAC and surgery at Pusan National University Hospital from 2015 to July 2022. The expression of the biomarker was assessed, and the overall survival (OS) and disease-free survival (DFS) were investigated. *Results*: After NAC, 24 patients (23.1%) out of 104 total patients had a pathological complete response (pCR). We found that changes in at least one biomarker were observed in 41 patients (51.2%), among 80 patients with RD. In patients with RD after NAC (n = 80), a subtype change was identified in 20 patients (25.0%). Any kind of change in the HER2 status was present 19 (23.7%) patients. The hormone receptor (HR)+/HER2+ subtype was significantly associated with better disease-free survival (DFS) (HR, 0.13; 95% CI, 0.02–0.99; *p* = 0.049). No change in p53 was associated with better DFS, and negative-to-positive change in p53 expression after NAC was correlated with worse DFS (*p* < 0.001). Negative-to-positive change in p53 was an independent, worse DFS factor in the multivariate analysis (HR,18.44; 95% CI, 1.86–182.97; *p* = 0.013). *Conclusions*: Biomarker change and subtype change after NAC were not infrequent, which can affect the further treatment strategy after surgery. The expression change of p53 might have a prognostic role. Overall, we suggest that the re-evaluation of biomarkers after NAC can provide a prognostic role and is needed for the best decision to be made on further treatment.

## 1. Introduction

Recently, neoadjuvant chemotherapy (NAC) has been considered a standard treatment for locally advanced breast cancer, and the use of NAC has increased [1].

Mohan et al. reported that the residual disease (RD) burden at the time of surgery after completion of NAC has been shown to have a significant effect on prognosis in all disease subtypes [2], but there is no agreement on the exact definition of RD or the pathological complete response (pCR). In the Miller–Payne grading system, the treatment response is estimated only as the reduction in primary invasive tumor cellularity and does not consider the presence or absence of ductal carcinoma in situ and lymph node metastasis [3]. In the residual cancer burden grading system, the treatment response is considered as the bidimensional diameter and cellularity of the invasive primary tumor, including lymph node metastasis, but the presence or absence of ductal carcinoma in situ is not considered [4]. The National Comprehensive Cancer Network (NCCN) guidelines, one of the most important guidelines, refers to pCR as no invasive and no in situ residual lesions in the breast and lymph nodes, and this concept can be used to best differentiate between patients with favorable and unfavorable outcomes [5]. Therefore, it is necessary to reach consensus on the evaluation criteria for the treatment response in patients with NAC to evaluate patient prognosis and establish a treatment standard in regard to NAC. One of the issues related to NAC is the biomarker status change. According to the NCCN guidelines, the biomarker status should be tested using the tumor core needle biopsy samples to determine the appropriate NAC, but it is not mandatory to repeat the biomarker status test using the resection samples to guide the adjuvant treatment choice. However, in prior studies, biomarker status was altered by NAC in some tumors. But biomarker status changes induced by NAC have been the focus of recent systemic and meta-analyses, and changes in the hormone receptor (HR) status induced by NAC can be used as a prognostic factor in breast cancer patients for predicting both overall survival (OS) and disease-free survival (DFS) [6,7]. Additionally, therapeutic options continue to be constrained in regard to pretreated advanced breast cancer patients, while several antibody–drug conjugates and immunotherapies are presently undergoing clinical trials [8,9]. Also, research is being conducted to investigate the effectiveness of combining various modalities in treatment and whether this effectiveness varies according to patient characteristics, such as gender [10]. It is anticipated that research will also explore which parameters will impact a patient’s treatment and investigate the most effective combination of therapeutic modalities tailored to individual patient characteristics.

However, there is relatively little published data on the impact of change in the receptor status on survival outcomes following neoadjuvant chemotherapy in breast cancer, but it should be considered in some circumstances [11]. In Korea, the data on biomarker changes after NAC and on the correlation between biomarker change and patient prognosis are limited. Thus, investigating biomarker discordance induced by NAC in Korean patients is important for determining treatment methods after surgery and predicting the patient’s progress.

The objective of this study was to explore biomarker discordance before and after NAC and to investigate ER, PR, HER2, p53, and Ki67 expression as a prognostic factor in RD patients after NAC.

## 2. Materials and Methods

### 2.1. Patients and Data Collection

We retrospectively evaluated 108 patients with invasive breast cancer who underwent surgery at Pusan National University Hospital from 2015 to July 2022. The patient was included if they: (1) received NAC prior to surgery, (2) underwent IHC biomarker testing on their core biopsy specimen at the time of diagnosis, (3) had paired immunohistochemistry biomarker testing on a surgical specimen. Among 108 patients, 4 patients were excluded because there was no paired testing. Clinical information was collected from electronic medical records, and pathologic information was collected from pathology reports. Exemption from informed consent after de-identification of the patients’ information was approved by the institutional review board at Pusan National University Hospital (2209-024-119).

### 2.2. Neoadjuvant Chemotherapy

All included patients had inoperable breast cancer, or HER2-positive or triple-negative disease, at an operable early-stage state. Also, they were treated with neoadjuvant chemotherapy (NAC) at Pusan National University Hospital. Patients with HER2-negative disease received 4 cycles of AC (doxorubicin, cyclophosphamide) every 21 days, followed by 4 cycles of paclitaxel or docetaxel. Patients with HER2-positive disease received 6 cycles of TCHP (docetaxel, carboplatin, trastuzumab, and pertuzumab) every 21 days. After NAC, all patients received breast conservative surgery or a mastectomy.

### 2.3. Immunohistochemistry

Immunohistochemistry for the estrogen receptor (ER; SP1, prediluted, Ventana Medical Systems, Tuscon, AZ, USA), progesterone receptor (PR; 1E2, prediluted, Ventana Medical Systems), human epidermal growth factor receptor type 2 (HER2; 4B5, prediluted, Ventana Medical Systems), p53 (DO7, prediluted, Ventana Medical Systems), and Ki67 (30-9, prediluted, Ventana Medical Systems) was performed using a Benchmark Ultra instrument (Ventana Medical Systems). The thickness of the paraffin sections was 3 μm. ER, PR, and HER2 positivity was assessed according to the American Society of Clinical Oncology (ASCO) guidelines. Staining for HRs (ER and PR) was considered positive when it exceeded 1% of any nuclear staining. HER2 positivity was defined as an immunostaining score of 3 (circumferential membrane staining that is complete, intense, and observed in >10% of tumor cells), or as gene amplification confirmed by silver in situ hybridization (SISH), which was performed with a HER2/CEP17 chromosome dual probe (Ventana Medical Systems). Cases with a HER2 immunostaining score of 2 (equivocal; weak-to-moderate complete membrane staining observed in >10% of tumor cells) received SISH testing to verify the HER2 gene amplification. A HER2 low positive case was defined as 1+ HER2 immunostaining or 2+ HER2 immunostaining without gene amplification on SISH. Positive staining for p53 was defined as strong diffuse nuclear staining, which is considered the most common pattern associated with mutations [12]. The Ki67 proliferation index was calculated as the overall average percentage of positive nuclear staining. If there were clear hot spots of Ki67 staining, data from these samples were also included in the overall score. The Ki67 proliferation index was categorized as low (<20%) or high (≥20%) for the purpose of the analysis. 

Patients were categorized into four subtypes based on their HR (ER and PR) and HER2 expression status: (1) HR+/HER2−, (2) HR+/HER2+, (3) HR−/HER2+, and (4) HR−/HER2−.

### 2.4. Evaluation of NAC Response

We used the strict definition of pCR according to the NCCN guidelines. We defined RD as any presence of an invasive or in situ lesion in the breast or lymph nodes, except if there was only a lympho-vascular invasion [5].

### 2.5. Statistics

We analyzed the discordance in the biomarker status between the biopsy specimen and the surgical specimen using the Chi-square test. Kaplan–Meier analysis was used to study prognosis (OS rate and DFS rate). OS was defined as the time from diagnosis to death or the last follow-up date, and DFS was defined as the time from diagnosis to any recurrence, new metastasis, and death. Multivariable Cox regression analysis was used to estimate the effects of the clinical and pathological variables. All analyses were performed using the SPSS statistical software (version 18; IBM, Armonk, NY, USA). All tests were two-sided and *p* < 0.05 was considered statistically significant.

## 3. Results

### 3.1. Patient Characteristics

The clinico-pathological characteristics of the patients included in this study are shown in Table 1. The median age of the patients at diagnosis was 54 years (range 24–77). Of the total 104 patients, 24 patients (23.1%) had a pCR, and 80 patients (76.9%) were in RD status after NAC. The subtypes of the biopsy samples pre-NAC were as follows: 33 patients (31.7%) were HR+/HER2−, 17 patients (16.4%) were HR+/HER2+, 28 patients (26.9%) were HR−/HER2+, and 26 patients (25.0%) were HR−/HER2−.

The pathological characteristics of RD patients (n = 80), according to pre-NAC biopsy subtypes, are presented in Table 2. In patients with RD after NAC (n = 80), a subtype change was identified in 20 patients (25.0%). Among them, a subtype change occurred in three (15.0%) patients with the HR+/HER2− biopsy subtype, eight patients (40.0%) with the HR+/HER2+ biopsy subtype, six patients (30.0%) with the HR−/HER2+ biopsy subtype, and three patients (15%) with the HR−/HER2− biopsy subtype.

### 3.2. Correlation between Clinico-Pathological Variables and NAC Response

The association between the clinico-pathological parameters and the NAC response is presented in Table 3. After NAC, 24 patients (23.1%) out of 104 total patients had a pCR. Their subtypes in regard to their biopsy samples before NAC are shown in Table 3. The pCR was most frequently observed in the HR−/HER2+ biopsy subtype (11, 45.8%) and at clinical stage III (20, 83.3%) (Table 3).

### 3.3. Changes in Each Biomarker Status after NAC

**Figure 1:**
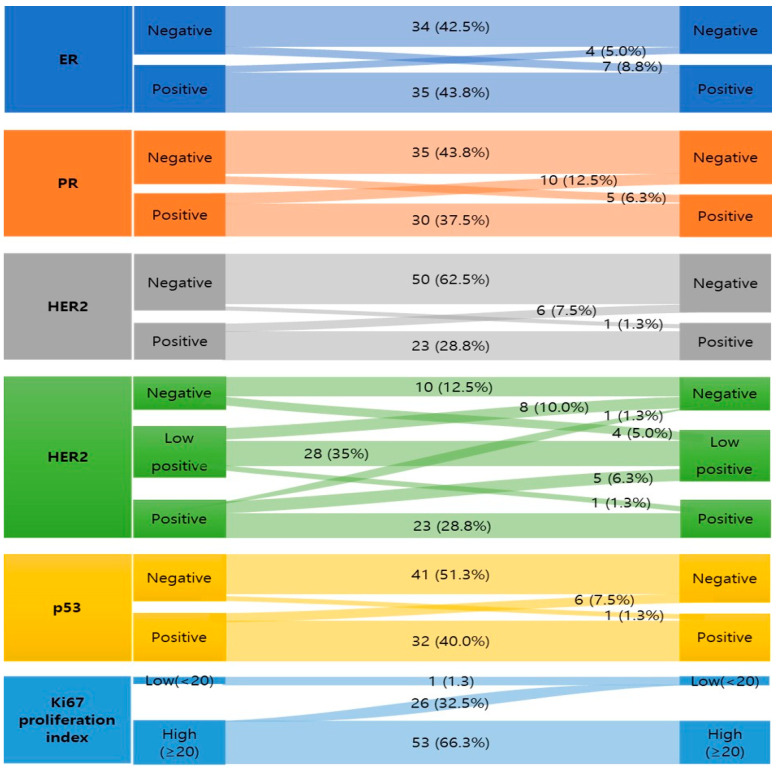
Biomarker changes after NAC (total residual patients, n = 80).

**Figure 2:**
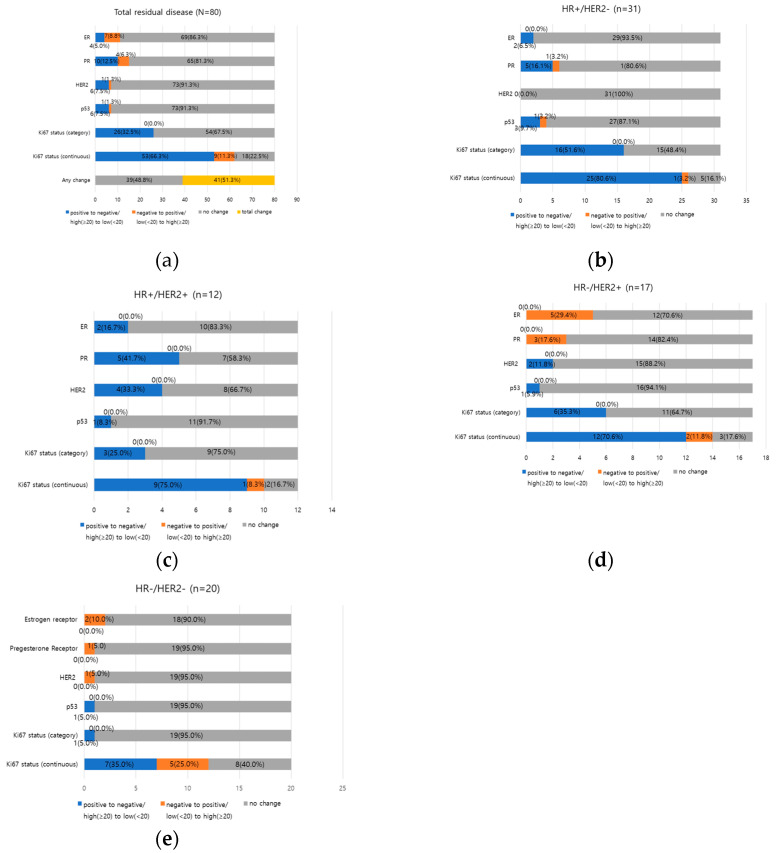
Biomarker changes after NAC according to biopsy subtype in residual disease patients (n = 80): (**a**) total residual patients (n = 80); (**b**) HR+/HER2− biopsy subtype (n = 31); (**c**) HR+/HER2+ biopsy subtype (n = 12); (**d**) HR−/HER2+ biopsy subtype (n = 17); (**e**) HR−/HER2− biopsy subtype (n = 20).

The changes in each biomarker status after NAC in 80 residual patients are shown in Figure 1. Most patients maintained stable expression of HRs, with 69 patients (86.3%) for ER and 65 patients (81.3%) for PR, as well as 61 patients (76.3%) for HER2 and 73 patients (91.3%) for p53. Additionally, 54 patients (67.6%) exhibited consistent Ki67 expression. However, of all 80 patients with RD, changes in at least one biomarker were observed in 41 patients (51.2%) (Figure 2a).

The biomarker status changes after NAC, according to the pre-NAC biopsy subtype, are presented in Figure 2b–e. The change in HER2 status, from positive to negative, was not identified in HR+/HER2− pre-NAC biopsy subtypes. Only positive to negative change in the HER2 status was identified in HER2+ subtypes (HR+/HER2+ and HR−/HER2+ subtypes). Notably, there was one case of negative to positive change in the HER2 status in HR−/HER2− subtypes and this case was a change from low positive to positive, which is described below (Figure 2e).

The HER2 status change, according to post-NAC resection subtypes, is presented in detail in Table 4. We found any kind of change in the HER2 status in 19 (23.7%) patients (Table 4). These changes include negative to low positive (4, 5.0%), low positive to negative (8, 10.0%), low positive to positive (1, 1.2%), positive to negative (1, 1.2%), and positive to low positive (5, 6.2%) (Table 4). Among them, five patients (6.2%) were HR+/HER2− subtype, four (5.0%) were HR+/HER2+ subtype, two (2.5%) were HR−/HER2+, and the remaining eight (10.0%) were HR−/HER2− subtype. Overall, there were six cases of positive to negative in terms of changes to the HER2 status.

The change in p53 expression after NAC was present in seven cases (8.7%). One (14.3%) of them had a negative to positive change and the other six patients (85.7%) showed a positive to negative change in p53 expression (Figure 1). The subtypes of the cases showing a p53 expression change are presented in Table 5.

The change in the Ki67 proliferation index group from low to high after NAC was not identified (Figure 1). However, in regard to the Ki-67 proliferation index as a continuous variable, there were nine cases (11.3%) of increment in the proliferation index; although it did not lead to a group change from low to high (Figure 2).

### 3.4. Overall Survival and Disease-Free Survival

**Figure 3:**
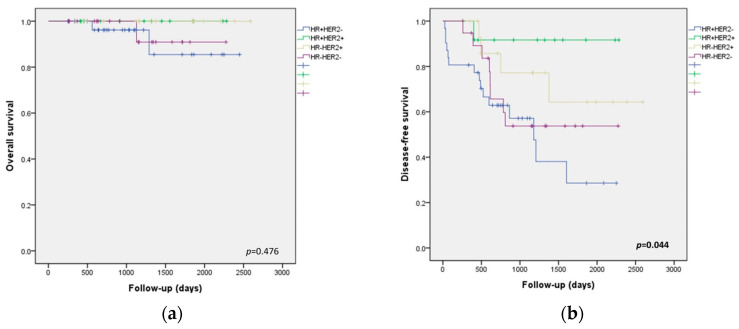

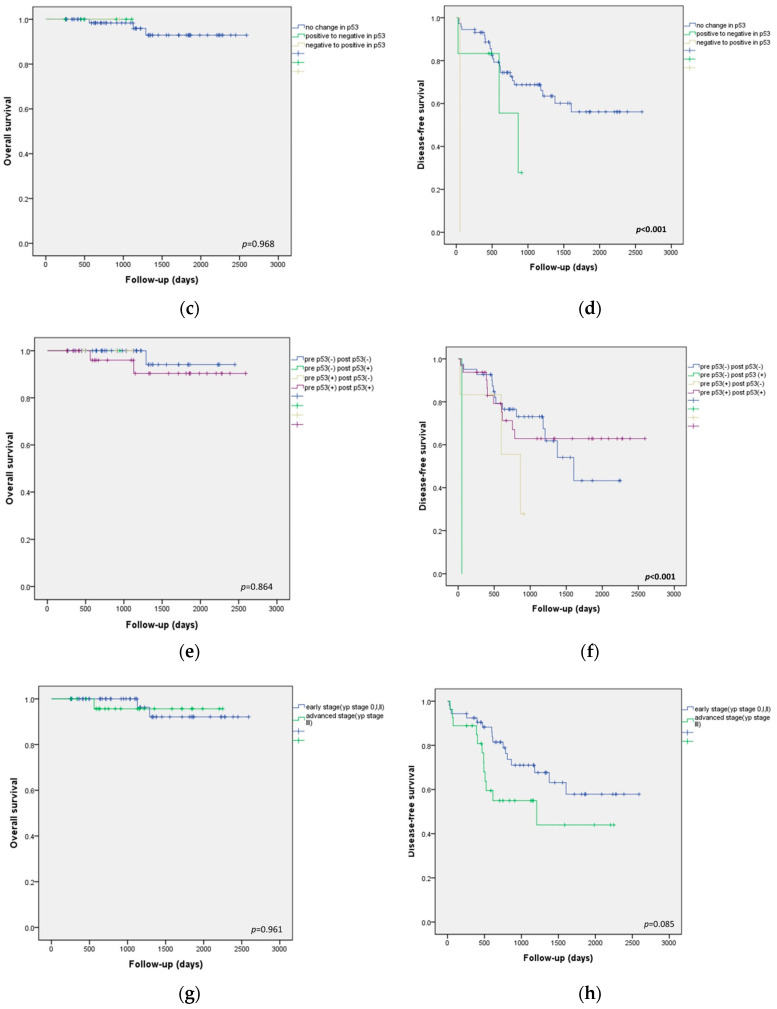
Kaplan–Meier estimates of overall survival and disease-free survival (residual disease patients, n = 80): (**a**) overall survival according to biopsy subtype (pre-NAC); (**b**) disease-free survival according to biopsy subtype (pre-NAC); (**c**) overall survival according to changes in p53 expression status after NAC; (**d**) disease-free survival according to changes in p53 expression status after NAC; (**e**) overall survival according to p53 status before and after NAC; (**f**) disease-free survival according to p53 status before and after NAC; (**g**) overall survival according to yp stage (post-NAC); (**h**) disease-free survival according to yp stage (post-NAC).

The survival analyses are shown in Figure 3. The HR+/HER2− pre-NAC biopsy subtype had the worst DFS, followed by the HR−/HER2− subtype. Also, the HR+/HER2+ subtype had the best DFS (*p* = 0.044, Figure 3b). The change in p53 expression had a significant impact on the DFS (*p* < 0.001). A negative to positive change in p53 had the worst DFS, while no change in p53 showed the best DFS (Figure 3d). According to the p53 status before and after NAC, no change in p53 was associated with better DFS; positivity of p53 in both pre-NAC biopsy and post-NAC resection samples had the best DFS, followed by negativity of p53 in both pre-NAC biopsy and post-NAC resection samples. Negative-to-positive change in p53 expression had the worst DFS (*p* < 0.001, Figure 3f). In addition, advanced yp stage (including yp stage III) patients after NAC tended to have worse DFS than early yp stage (including yp stage 0, I, II) patients (*p* = 0.085, Figure 3h).

In the multivariate analysis, the HR+/HER2+ subtype was an independent factor for better DFS (HR, 0.13; 95% CI, 0.02–0.99; *p* = 0.049). Also, negative-to-positive change in p53 expression after NAC was an independent factor for worse DFS (HR, 18.44; 95% CI, 1.86–182.97; *p* = 0.013) (Table 6).

## 4. Discussion

We found that biomarker change was common (51.2% of the RD patients) between specimens taken before and after NAC. A change in p53 expression after NAC was associated with a particularly poor prognosis in RD patients.

Biomarker change, according to NAC, in breast cancer patients appears in various ways, according to previous studies. Changes in biomarkers after NAC in breast cancer patients have been studied mainly in regard to Ki-67 [13,14,15] and it usually changes after adjusting the NAC [16,17]. Ki-67, a marker of cell proliferation, is expressed in all phases of the cell cycle, except G0 [18]. A decrease in Ki-67 after NAC was associated with a pCR and with better DFS and OS [13,15]. Rey-Vargas et al. observed no significant correlation between a Ki-67 decrease and the survival rate, but they reported a tendency of Ki-67 to decrease after NAC [14]. The same was true for this study, in which 26 patients (32.5%) showed a discordance of Ki-67 between pre-NAC and post-NAC specimens and all changes involved a decline, but these changes were not significantly correlated with the survival rate.

HER2 has been used as a treatment target over the past few decades since trastuzumab was developed. Recently, early-phase clinical trials have reported promising antiHER2 antibody–drug conjugates (ADCs), trastuzumab–deruxtecan and trastuzumab–duocarmazine, in HER2 low-positive patients [19,20]. Ahn et al. reported that positive-to-negative change in HER2 expression was more common than negative-to-positive change after NAC [21]. Also, Tural et al. showed that HER2 status change from positive to negative was an independent risk factor for worse DFS [22]. In this study, we did not reveal HER2 change as a prognostic factor after NAC. However, any change that can alter further treatment after NAC resection was significant in number, namely 19 patients (23.7%). Of note, four patients had negative HER2 expression in their pre-NAC biopsy sample and then showed HER2 low positive in their resection specimen after NAC. While it has not yet been established as the standard treatment protocol, the transition to HER2 low-positive status in patients following therapy suggests the potential diversification of treatment modalities in the future.

TP53, which encodes for the tumor suppressor protein p53, is the most frequently mutated gene in most types of human cancer, including breast cancer [23]. The role of p53 as a prognostic factor predicting pCR after NAC is controversial [24,25,26]. Bae et al. investigated, regardless of p53 expression, before NAC; the high expression of the p53 group after NAC indicated better OS in triple-negative breast cancer (TNBC) patients receiving NAC [27].

We found that no change in p53 expression after NAC was a significant predictor of improved prognosis. In particular, p53 positivity in both pre-NAC and post-NAC specimens was associated with the best improved prognosis. A few studies have shown that p53 positivity predicted chemotherapy-sensitive disease compared with p53 negative cases in TNBC [26,27]. In our study, patients with p53 positivity, both pre-NAC and post-NAC, had a better prognosis than those with negative p53 in both specimens.

In addition, we observed that any change in the p53 status was associated with worse prognosis than no change. In particular, a negative-to-positive change in p53 expression after NAC predicted a lower DFS. In patients who did not achieve a pCR, NAC resulted in a subpopulation of chemotherapy-resistant cells [28]. And diffuse nuclear positivity for p53 has previously been shown to be highly correlated with TP53 mutations [29]. Balko et al. molecularly profiled the RD remaining after NAC in a cohort of 111 TNBC. Alterations in TP53 were identified in 89% of the samples [30]. MCL1 gene amplifications were seen in 54%, and MYC gene amplifications were seen in 35% of the samples [30]. These findings suggest that these alterations are present at high frequency in chemotherapy-treated TNBCs and may play a role in de novo or acquired therapeutic resistance [30]. Therefore, although this study was not limited to the triple-negative subtype, we could consider the possibility of the emergence of treatment resistance in patients with a p53 status alteration.

Patients with HR+ subtypes of breast cancer have the best prognosis, by contrast patients with HR− subtypes, especially those with triple-negative disease, have the worst prognosis, in part because of the lack of a receptor target [31,32]. We also observed that HR+/HER2+ patients who underwent NAC had improved DFS, in the multivariable analysis. Interestingly, however, among patients with NAC, the HR+HER2− subtype had worse prognosis than the HR−HER2− subtype. Luminal A breast cancers generally have a good prognosis and respond well to hormonal therapies, and patients with these cancers do not appear to benefit from the addition of the microtubule-targeted chemotherapy drug paclitaxel commonly used for NAC [33]. This may explain the poor therapeutic effect of NAC in luminal A breast cancer patients. In addition to this, after NAC, the residual tumors of most such patients had alterations in at least one of the clinically targetable pathways, resulting in therapeutic resistance [30]. These are the likely reasons why luminal A breast cancer patients had the worst prognosis in this study.

This study had several limitations. Firstly, it was conducted retrospectively, and this suggests the potential presence of confounding factors that were not considered. Secondly, the sample size was relatively small and data collection was carried out in a single institution. Thirdly, recent studies suggest that the distribution of residual disease, whether scattered or concerted, may impact patients’ long-term survival [34,35]. Nevertheless, the distribution pattern of residual tumors was not considered in this study. However, it is important that the collected data can be used as a foundation for further research and provide insight on biomarker changes after NAC.

## 5. Conclusions

Our findings suggested that NAC has the potential to elicit alterations in biomarker expression and ultimately results in a subtype change after NAC, which was not common. In addition, p53 expression change may provide a prognostic role. Further studies are needed to clarify these issues and determine the need to re-evaluate biomarkers after NAC. This small and retrospective study provides a basis for future research investigating the prognostic and predictive role of biomarker re-evaluation.

## Figures and Tables

**Table 1:** Clinico-pathological characteristics of study cohort.

**Age at Diagnosis**		**54 (24–77)**	Total patients (n = 104)
NAC response	Pathological complete response (pCR)	24 (23.1)	
	Residual disease (RD)	80 (76.9)	
Pre-NAC subtypes (biopsy)	HR+/HER2−	33 (31.7)	
	HR+/HER2+	17 (16.4)	
	HR−/HER2+	28 (26.9)	
	HR−/HER2−	26 (25.0)	
Pre-NAC clinical stage (c stage, biopsy)	I	3 (2.9)	
	II	17 (16.3)	
	III	79 (76.0)	
	IV	5 (4.8)	
Pre-NAC nuclear grade (biopsy)	Low (grade 1, 2)	71 (68.3)	
	High (grade 3)	29 (27.9)	
	Not applicable	4 (3.8)	
Post-NAC pathological stage (yp stage, resection)	0	32 (30.8)	
	I	14 (13.5)	
	II	31 (29.8)	
	III	27 (26.0)	
Post-NAC lympho-vascular invasion (resection)	Absent	72 (69.2)	
	Present	32 (30.8)	
Post-NAC nuclear grade (resection)	Low (grade 1, 2)	35 (33.7)	
	High (grade 3)	33 (31.7)	
Post-NAC histological grade (resection)	Not applicable	36 (34.6)	
	Low (well, moderately)	39 (37.5)	
	High (poorly)	28 (26.9)	
Post-NAC subtypes (resection)	Not applicable	37 (35.6)	Residual disease patients (n = 80)
	HR+/HER2−	36 (45.0)	
	HR+/HER2+	7 (8.8)	
	HR−/HER2+	16 (20.0)	

**Table 2:** Pathologic characteristics of residual disease patients, according to pre-NAC biopsy subtypes (n = 80).

		Pre-NAC Subtypes (Biopsy)			
		HR+/HER2− (n = 31)	HR+/HER2+ (n = 12)	HR−/HER2+ (n = 17)	HR−/HER2− (n = 20)
Pre-NAC nuclear grade (biopsy)	Low (grade 1, 2)	25 (80.6)	11 (91.7)	7 (41.2)	11 (55.0)
	High (grade 3)	4 (12.9)	1 (8.3)	10 (58.8)	8 (40.0)
	Not applicable	2 (6.5)	0 (0.0)	0 (0.0)	1 (5.0)
Pre-NAC clinical stage (c stage, biopsy)	I	3 (9.7)	0 (0.0)	0 (0.0)	0 (0.0)
	II	6 (19.4)	2 (16.7)	3 (17.6)	2 (10.0)
	III	20 (64.5)	9 (75.0)	13 (76.5)	17 (85.0)
	IV	2 (6.5)	1 (8.3)	1 (5.9)	1 (5.0)
Post-NAC subtypes (resection)	HR+/HER2−	28 (90.3)	4 (33.3)	2 (11.8)	2 (10.0)
	HR+/HER2+	0 (0.0)	4 (33.3)	3 (17.6)	0 (0.0)
	HR−/HER2+	0 (0.0)	4 (33.3)	11 (64.7)	1 (5.0)
	HR−/HER2−	3 (9.7)	0 (0.0)	1 (5.9)	17 (85.0)
Post-NAC nuclear grade (resection)	Low (grade 1)	22 (71.0)	4 (33.3)	6 (35.3)	3 (15.0)
	High (grade 2, 3)	6 (19.4)	7 (58.3)	6 (35.3)	14 (70.0)
	Not applicable	3 (9.7)	1 (8.3)	5 (29.4)	3 (15.0)
Post-NAC pathological stage (yp stage, resection)	0	3 (9.7)	1 (8.3)	3 (17.6)	1 (5.0)
	I	5 (16.1)	2 (16.7)	6 (35.3)	1 (5.0)
	II	10 (32.3)	8 (66.7)	2 (11.8)	11 (55.0)
	III	13 (41.9)	1 (8.3)	6 (35.3)	7 (35.0)

**Table 3:** Correlation between NAC response and pathologic variables in total patients (n = 104).

Variables		Pathological Complete Response (n = 24)	Residual Disease (n = 80)	*p*-Value
Age at Diagnosis		55.5 Years (29–71)	54 Years (24–77)	
Pre-NAC subtypes (biopsy)	HR+/HER2−	2 (8.3)	31 (38.8)	0.019
	HR+/HER2+	5 (20.8)	12 (15.0)	
	HR−/HER2+	11 (45.8)	17 (21.3)	
	HR−/HER2−	6 (25.0)	20 (25.0)	
Pre-NAC clinical stage (c stage, biopsy)	I	0 (0.0)	3 (3.8)	0.453
	II	4 (16.7)	13 (16.3)	
	III	20 (83.3)	59 (73.8)	
	IV	0 (0.0)	5 (6.3)	
Pre-NAC nuclear grade (biopsy)	Low (grade 1, 2)	17 (70.8)	54 (67.5)	0.926
	High (grade 3)	6 (25.0)	23 (28.8)	
	Not applicable	1 (4.2)	3 (3.8)	
Post-NAC pathological stage (yp stage, resection)	0	24 (100.0)	8 (10.0)	<0.001
	I	0 (0.0)	14 (17.5)	
	II	0 (0.0)	31 (38.8)	
	III	0 (0.0)	27 (33.8)	
Post-NAC lympho-vascular invasion (resection)	Absent	23 (95.8)	49 (61.3)	0.001
	Present	1 (4.2)	31 (38.8)	
Post-NAC nuclear grade (resection)	Low (grade 1, 2)	0 (0.0)	35 (43.8)	<0.001
	High (grade 3)	0 (0.0)	33 (41.3)	
	Not applicable	24 (100.0)	12 (15.0)	
Post-NAC histological grade (resection)	Low (well, moderately)	0 (0.0)	39 (48.8)	<0.001
	High (poorly)	0 (0.0)	28 (35.0)	
	Not applicable	24 (100.0)	13 (16.3)	

**Table 4:** HER2 expression changes according to post-NAC subtypes in residual disease patients (n = 80).

		Post-NAC Subtypes (Resection)																			
		Total Residual (n = 80)				HR+/HER2− (n = 31)				HR+/HER2+ (n = 12)				HR−/HER2+ (n = 17)				HR−/HER2− (n = 20)			
		Negative	Low Positive	Positive	*p*-Value	Negative	Low Positive	Positive	*p*-Value	Negative	Low Positive	Positive	*p*-Value	Negative	Low Positive	Positive	*p*-Value	Negative	Low Positive	Positive	*p*-Value
Pre-NAC HER2 status (biopsy)	Negative	10 (52.6)	4 (10.8)	0 (0.0)	<0.001	5 (62.5)	2 (8.7)	0 (0.0)	0.002	0 (0.0)	0 (0.0)	0 (0.0)		0 (0.0)	0 (0.0)	0 (0.0)		5 (50.0)	2 (22.2)	0 (0.0)	0.337
	Low positive	8 (42.1)	28 (75.7)	1 (4.2)		3 (37.5)	21 (91.3)	0 (0.0)		0 (0.0)	0 (0.0)	0 (0.0)		0 (0.0)	0 (0.0)	0 (0.0)		5 (50.0)	7 (77.8)	1 (100.0)	
	Positive	1 (5.3)	5 (13.5)	23 (95.8)		0 (0.0)	0 (0.0)	0 (0.0)		1 (100.0)	3 (100.0)	8 (100.0)		0 (0.0)	2 (100.0)	15 (100.0)		0 (0.0)	0 (0.0)	0 (0.0)	

(a) HER2 expression in immunohistochemistry (IHC) 0; (b) HER2 expression in IHC 1+ or 2+ in the absence of HER2 gene amplification; (c) HER2 expression in IHC 2+ in the presence of HER2 gene amplification or IHC 3.

**Table 5:** Cases showing p53 expression change after NAC.

Case	Change Type	Pre-NAC Biopsy Subtype	Post-NAC Resection Subtype
5	Positive to negative	HR+/HER2−	HR+/HER2−
13	Negative to positive	HR+/HER2−	HR+/HER2−
19	Positive to negative	HR+/HER2+	HR+/HER2−
20	Positive to negative	HR−/HER2+	HR+/HER2+
39	Positive to negative	HR−/HER2−	HR−/HER2−
82	Positive to negative	HR+/HER2−	HR+/HER2−
91	Positive to negative	HR+/HER2−	HR−/HER2−

**Table 6:** Analysis of disease-free survival in residual disease patients (n = 80).

		Univariable		Multivariable	
		Hazard Ratio (95% CI)	*p*-Value	Hazard Ratio (95% CI)	*p*-Value
Pre-NAC subtypes (biopsy)	HR+/HER2−	1	0.086	1	0.131
	HR+/HER2+	0.12 (0.02–0.90)	0.039	0.13 (0.02–0.99)	0.049
	HR−/HER2+	0.37 (0.12–1.12)	0.079	0.41 (0.13–1.25)	0.115
	HR−/HER2−	0.69 (0.29–1.62)	0.387	0.74 (0.31–1.76)	0.49
Pre-NAC clinical stage (c Stage, biopsy)	Early (0, I, II)	1			
	Advanced (III, IV)	1.71 (0.59–4.94)	0.319		
Post-NAC pathologic stage (yp Stage, resection)	Early (0, I, II)	1			
	Advanced (III)	1.92 (0.90–4.08)	0.09		
Post-NAC histologic grade (resection)	Low (well, moderately) High (poorly) Not applicable	0.75 (0.29–1.99)	0.567		
		0.59 (0.21–1.66)	0.314		
		1	0.600		
ER change	No change	1	0.754		
	Positive to negative	1.44 (0.34–6.13)	0.620		
	Negative to positive	0.68 (0.16–2.88)	0.600		
PR change	No change	1	0.665		
	Positive to negative	1.63 (0.56–4.75)	0.371		
	Negative to positive	0.98 (0.23–4.17)	0.977		
HER2 change	No change	1	0.563		
	Positive to negative	0	0.978		
	Negative to positive	3.00 (0.40–22.46)	0.284		
P53 change	No change	1	0.008	1	0.03
	Positive to negative	2.34 (0.70–7.90)	0.170	1.972 (0.58–6.67)	0.275
	Negative to positive	28.26 (2.92–273.57)	0.004	18.44 (1.86–182.97)	0.013
Ki67 change	No change	1			
	High (≥20) to low(<20)	0.79 (0.35–1.80)	0.577		
	Low (<20) to high(≥20)				

## Data Availability

The data presented in this study are available on request from the corresponding author due to ethical reasons.
